# Supplementary material for: Genomic Insights into Idiopathic Granulomatous Mastitis through Whole-Exome Sequencing: A Case Report of Eight Patients
Source: Int J Mol Sci. 2024 Aug 21;25(16):9058. doi: 10.3390/ijms25169058 (PMC11354296; doi:10.3390/ijms25169058)
Supplement: Supplementary file 1 [file ijms-25-09058-s001.zip › ijms-3158850-supplementary.pdf]

Genomic Insights into Idiopathic Granulomatous Mastitis through Whole-Exome Sequencing: A Case Report of Eight Patients

Supplementary Materials

Item	Title	Main text reference	Supplementary materials page number
Supplementary Table S1	Concentration and yield of extracted genomic DNA before whole-exome sequencing (WES) library preparation.	Page 3	2
Supplementary Figure S1	Yield of extracted genomic DNA before whole-exome sequencing (WES) library preparation.	Page 3	3
Supplementary Table S2	Concentration and yield from whole-exome sequencing (WES) library preparation.	Page 3	4
Supplementary Table S3	Summary statistics of sequencing performance, coverage metrics and sequencing reads quality control values.	Page 3<br>Page 4	5
Supplementary Table S4	Somatic variants identified from whole-exome sequencing (WES) of blood samples through *Strelka2* and *Mutect2* variant calling.	Page 4<br>Page 8	7
Supplementary Table S5	*EnrichR* functional enrichment of genes altered in protein-truncating variants (PTVs) called by *Strelka2* and *Mutect2* matched blood-tissue variant calling, using 2019 version of Kyoto Encyclopedia of Genes and Genomes (KEGG) knowledge base.	Page 7	8
Supplementary Figure S2	Visualisation of enriched pathways from Supplementary Table 5, ordered by *p*-value.	Page 7	10
Supplementary Table S6	Somatic variants identified from whole-exome sequencing (WES) of blood samples that overlap across *Strelka2* and *Mutect2* variant calling.	Page 8	11
Supplementary Table S7	Variants identified through whole-exome sequencing (WES) validation with Sanger sequencing.	Page 8	12

Supplementary Table S1. Concentration and yield of extracted genomic DNA before whole-exome sequencing (WES) library preparation.

Case	Sample	Nucleic Acid	Unit	Yield (ng)
1	Tissue	9.6	ng/µl	480
2	Tissue	13.4	ng/µl	670
3	Tissue	12.5	ng/µl	625
4	Tissue	13.6	ng/µl	680
5	Tissue	13	ng/µl	650
6	Tissue	13.4	ng/µl	670
7	Tissue	13	ng/µl	650
8	Tissue	11.6	ng/µl	580
1	Blood	12.1	ng/µl	605
2	Blood	11	ng/µl	550
3	Blood	10.5	ng/µl	525
4	Blood	9.8	ng/µl	490
5	Blood	9.7	ng/µl	485
6	Blood	8.4	ng/µl	420
7	Blood	10.9	ng/µl	545
8	Blood	13	ng/µl	650

Supplementary Figure S1. Yield of extracted genomic DNA before whole-exome sequencing (WES) library preparation.

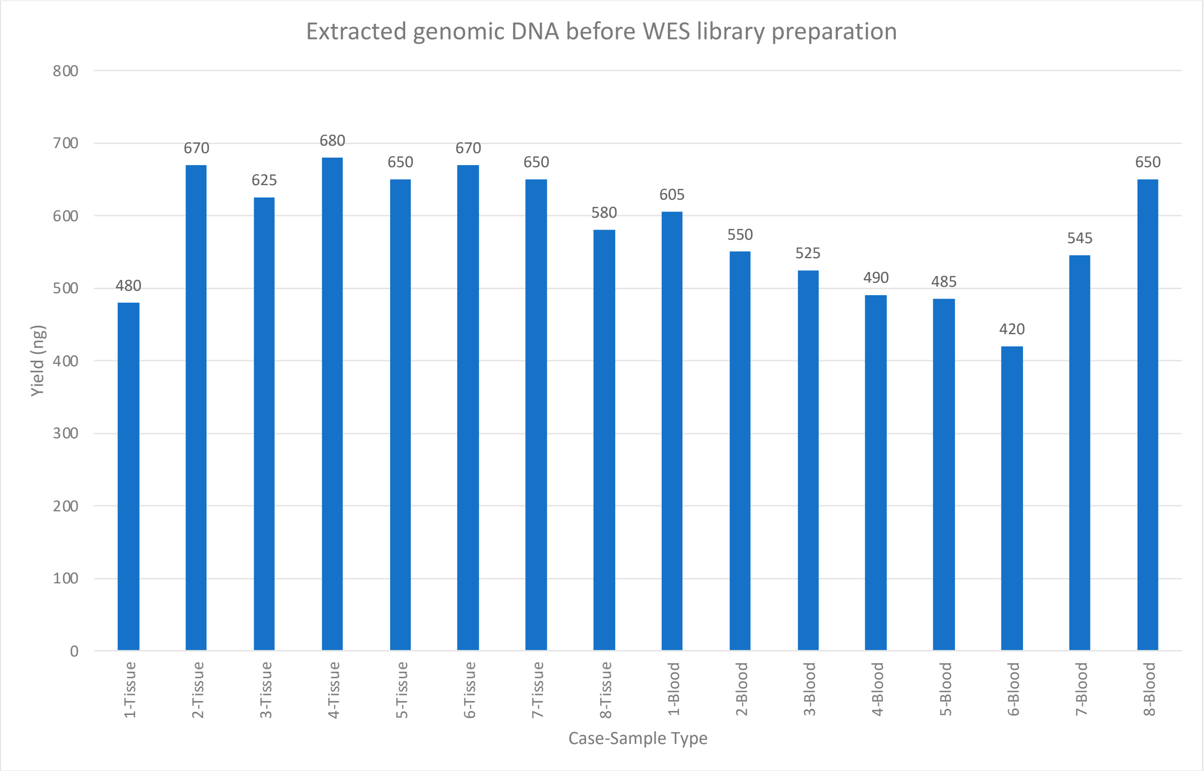

Supplementary Table S2. Concentration and yield from whole-exome sequencing (WES) library preparation.

Case	Sample Type	Concentration (ng/ul)	Concentration (nM)	Peak (bp)	Yield (ng)
1	Tissue	3.83	16.3	315	191.5
2	Tissue	7.42	31	324	371
3	Tissue	8.59	35.9	332	429.5
4	Tissue	7.2	31.5	322	360
5	Tissue	9.45	41.9	315	472.5
6	Tissue	9.58	40.8	320	479
7	Tissue	9.52	41.6	320	476
8	Tissue	11.1	47.5	318	555
1	Blood	11.5	48.2	329	575
2	Blood	11.7	49.6	323	585
3	Blood	5.13	21.5	328	256.5
4	Blood	5.52	23.1	329	276
5	Blood	5.47	22.7	337	273.5
6	Blood	5.15	21.6	335	257.5
7	Blood	6.01	25.6	320	300.5
8	Blood	6.24	26.5	325	312

Supplementary Table S3. Summary statistics of sequencing performance, coverage metrics and sequencing reads quality control values.

Case-Sample Type	Yield (Mbases[1], thousands)	Total reads (millions)	% PF[2] clusters	% >= Q30 bases[3]	Mean Quality score	Mapping efficiency (%)	Duplicate reads (%)	Mean Coverage[4] Depth (x)	% coverage region[4] >20x	% GC content[5]
1-Tissue	13,271	88	100	68.45	32.08	99.99819302	13.60571419	34.71	100	42.4
2-Tissue	15,912	106	100	81.79	35.45	99.99896609	16.5703934	44.46	100	43.5
3-Tissue	17,606	117	100	70.38	32.57	99.99864224	14.22206515	45.49	100	42.3
4-Tissue	17,631	118	100	79.45	34.85	99.99898396	16.39147259	48.09	100	43.2
5-Tissue	17,299	115	100	75.64	33.87	99.99889577	16.91178391	47.93	100	43.2
6-Tissue	19,107	127	100	83.61	35.91	99.99904566	19.87942732	53.51	100	43.4
7-Tissue	15,278	102	100	78.35	34.5	99.99903568	16.54351951	43.61	100	43.3
8-Tissue	13,276	89	100	84	36.01	99.99897287	18.30835869	38.6	100	43.7
1-Blood	10,478	70	100	81.34	35.33	99.99853774	17.7817895	26.66	100	43.4
2-Blood	11,066	74	100	82.23	35.56	99.99812482	18.11617778	30.99	100	43.3
3-Blood	11,759	78	100	81.35	35.34	99.99785608	18.88808341	31.93	100	43.3
4-Blood	9,728	65	100	74.34	33.55	99.997887	14.37341294	26.75	100	42.9
5-Blood	10,760	72	100	78.97	34.71	99.99815122	18.65911251	30.26	100	43.2
6-Blood	11,824	79	100	73.42	33.31	99.99768945	17.99989554	32.36	100	42.9
7-Blood	11,219	75	100	81.88	35.46	99.99816468	18.52028221	31.85	100	43.4
8-Blood	11,659	78	100	82.39	35.59	99.99817696	17.9521995	32.78	100	43.2
Mean (Tissue)	16,172.5	107.8	100.0	77.7	34.4	100.0	16.6	44.6	100	43
Mean (Blood)	11,061.6	73.7	100.0	79.5	34.9	100.0	17.8	30.4	100	43

Mean (All samples)	13,617.1	90.8	100.0	78.6	34.6	100.0	17.2	37.5	100	43

[1]Total number of megabases (millions of bases) of DNA sequenced, reported in thousands. This measures the output of a sequencing run.
[2]Percentage of clusters passing filter: Proportion of clusters (groups of DNA sequences) that pass quality filters during sequencing.
[3]Percentage of bases with a quality score of 30 or higher. High percentages suggest high-quality sequencing data.
[4]Coverage depth and coverage region corresponds to the target exonic regions.
[5]Percentage of guanine (G) and cytosine (C) bases in the DNA sequence.

Supplementary Table S4. Somatic variants identified from whole-exome sequencing (WES) of blood samples through *Strelka2* and *Mutect2* variant calling.

Case	Somatic variants		SNVs[1]		Indels[2]		PTVs[3]		Pathogenic[4]		Pathogenic/Likely Pathogenic[4]		Likely Pathogenic[4]	
	Strelka2	***Mutect2***	***Strelka2***	***Mutect2***	***Strelka2***	***Mutect2***	***Strelka2***	***Mutect2***	***Strelka2***	***Mutect2***	***Strelka2***	***Mutect2***	***Strelka2***	***Mutect2***
1	7727	696	5915	677	1812	19	95	44	1	1	0	0	0	0
2	6075	794	4518	786	1557	8	61	50	0	0	0	0	0	0
3	6089	959	4592	951	1497	8	62	66	0	1	0	0	0	0
4	11947	614	9992	605	1955	9	141	53	0	1	0	0	0	1
5	6387	840	4716	825	1671	15	72	60	1	1	0	1	0	0
6	7328	968	5630	957	1698	11	75	70	1	3	0	0	1	1
7	5391	732	3798	723	1593	9	56	43	1	1	0	0	0	0
8	5821	891	4412	878	1409	13	58	59	0	1	0	1	0	0
Median (range)	6238 (5391-11947)	817 (614-968)	4654 (3798-9992)	805.5 (605-957)	1632(1409-1955)	10 (8-19)	67 (56-141)	56 (43-70)	0.5 (0-1)	1 (0-3)	0 (0-0)	0 (0-1)	0 (0-1)	0 (0-1)

[1] Single nucleotide variants
[2] Insertions and deletions
[3] Protein-truncating variants. These correspond to variants annotated as nonsense mutations, or frameshift insertions or deletions by GATK4 *Funcotator*.
[4] *ClinVar* annotation of pathogenicity within GATK4 *Funcotator* variant annotation.

Supplementary Table S5. *EnrichR* functional enrichment of genes altered in protein-truncating variants (PTVs) called by *Strelka2* and *Mutect2* matched blood-tissue variant calling, using 2019 version of Kyoto Encyclopedia of Genes and Genomes (KEGG) knowledge base.

**Enriched pathway**	**Overlap**	***p*-value**	**Adjusted *p*-value**	**Odds ratio**	**Combined score**	**Genes**
Terpenoid backbone biosynthesis	1/22	0.0526	0.481	19.772	58.242916	IDI2
Protein export	1/23	0.0549	0.481	18.872	54.7763046	SEC62
Protein processing in endoplasmic reticulum	2/165	0.0618	0.481	5.166	14.3790161	DNAJB12; SEC62
Huntington disease	2/193	0.0811	0.481	4.402	11.0573367	DNAH1; DNAH9
Vasopressin-regulated water reabsorption	1/44	0.1024	0.481	9.645	21.9790026	DYNC1I2
ABC transporters	1/45	0.1046	0.481	9.426	21.2777177	ABCB5
Regulation of lipolysis in adipocytes	1/55	0.1264	0.481	7.676	15.8788045	PTGER3
Adherens junction	1/72	0.1622	0.481	5.833	10.6117607	FER
Salmonella infection	1/86	0.1906	0.481	4.869	8.07205861	DYNC1I2
Protein digestion and absorption	1/90	0.1985	0.481	4.649	7.51796421	COL14A1
mRNA surveillance pathway	1/91	0.2005	0.481	4.597	7.38859473	SMG1
Toll-like receptor signaling pathway	1/104	0.2257	0.481	4.015	5.97620039	IFNAR1
Osteoclast differentiation	1/127	0.2684	0.481	3.278	4.31143448	IFNAR1
Natural killer cell mediated cytotoxicity	1/131	0.2756	0.481	3.176	4.09396586	IFNAR1
Estrogen signaling pathway	1/137	0.2862	0.481	3.035	3.79702497	FKBP4
Measles	1/138	0.2880	0.481	3.013	3.75064154	IFNAR1
Phagosome	1/152	0.3122	0.481	2.732	3.18001247	DYNC1I2
Hepatitis C	1/155	0.3173	0.481	2.678	3.07435193	IFNAR1
Cellular senescence	1/160	0.3257	0.481	2.593	2.90925752	HIPK3
JAK-STAT signaling pathway	1/162	0.3290	0.481	2.561	2.84680162	IFNAR1
Necroptosis	1/162	0.3290	0.481	2.561	2.84680162	IFNAR1
Hepatitis B	1/163	0.3307	0.481	2.545	2.81629828	IFNAR1
Influenza A	1/171	0.3438	0.481	2.424	2.58838978	IFNAR1
NOD-like receptor signaling pathway	1/178	0.3550	0.481	2.327	2.41007428	IFNAR1
Axon guidance	1/181	0.3598	0.481	2.288	2.33899979	ROBO1
Kaposi sarcoma-associated herpesvirus infection	1/186	0.3677	0.481	2.226	2.2270141	IFNAR1
Calcium signaling pathway	1/188	0.3708	0.481	2.202	2.18434688	PTGER3
Pathways in cancer	2/530	0.3742	0.481	1.565	1.53879087	PTGER3; IFNAR1
Epstein-Barr virus infection	1/201	0.3908	0.485	2.057	1.93327146	IFNAR1
cAMP signaling pathway	1/212	0.4071	0.489	1.949	1.75139935	PTGER3
Human cytomegalovirus infection	1/225	0.4260	0.495	1.835	1.56580724	PTGER3
Cytokine-cytokine receptor interaction	1/294	0.5164	0.581	1.398	0.92370317	IFNAR1
Human papillomavirus infection	1/330	0.5579	0.600	1.243	0.72509741	IFNAR1
Neuroactive ligand-receptor interaction	1/338	0.5666	0.600	1.213	0.68875736	PTGER3

PI3K-Akt signaling pathway	1/354	0.5836	0.600	1.157	0.62287812	IFNAR1
Herpes simplex virus 1 infection	1/492	0.7053	0.705	0.826	0.28822203	IFNAR1

Supplementary Figure S2. Visualisation of enriched pathways from Supplementary Table 5, ordered by *p*-value.

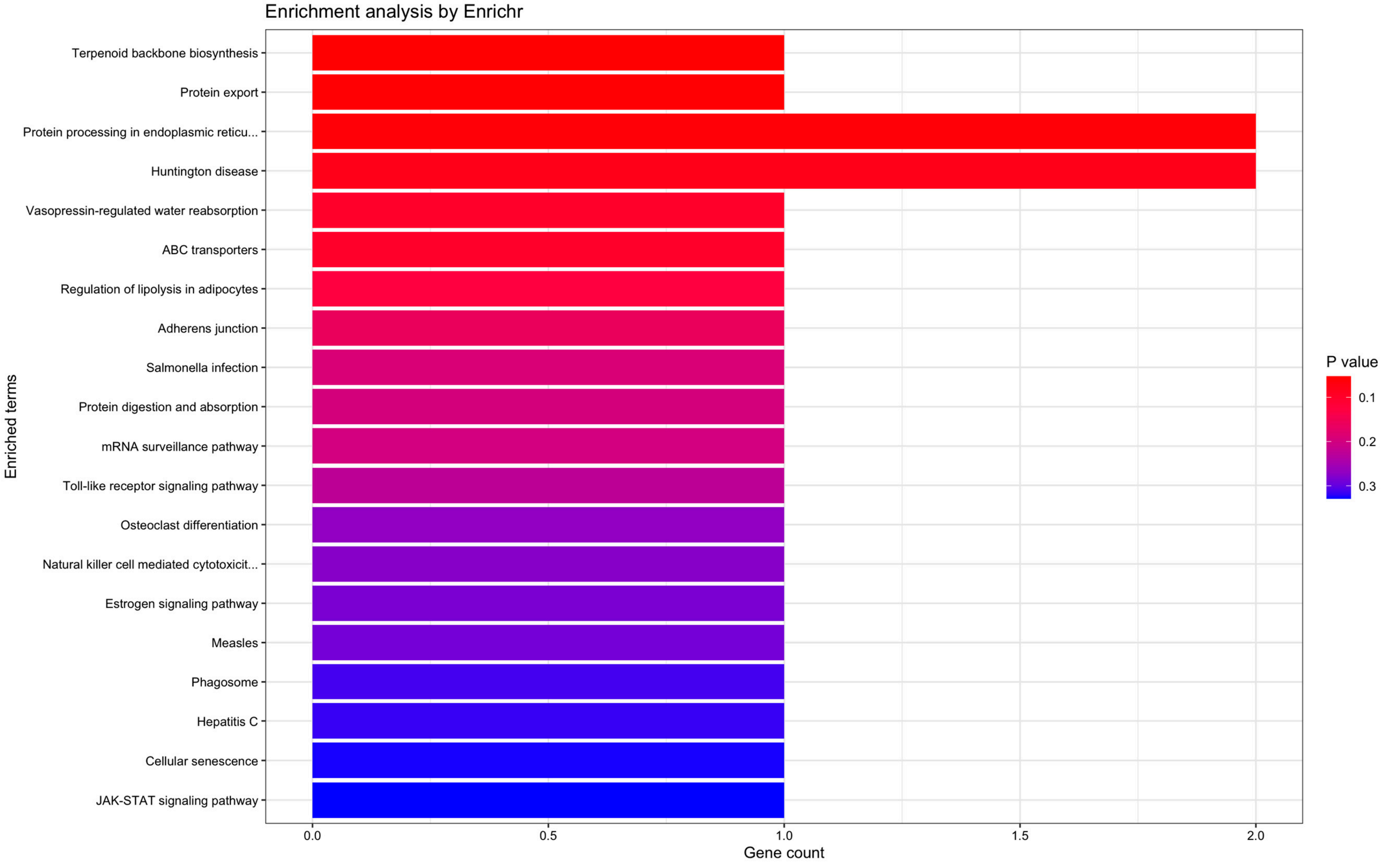

Supplementary Table S6. Somatic variants identified from whole-exome sequencing (WES) of blood samples that overlap across *Strelka2* and *Mutect2* variant calling.

Case	Somatic variants	SNVs[1]	Indels[2]	PTVs[3]	Pathogenic[4]	Pathogenic / Likely Pathogenic[4]	Likely Pathogenic[4]
1	37	32	5	0	0	0	0
2	73	62	11	2	0	0	0
3	74	67	7	3	0	0	0
4	78	66	12	1	0	0	0
5	68	58	10	1	0	0	0
6	81	63	18	2	0	0	0
7	57	46	11	2	0	0	0
8	74	62	12	1	0	0	0
Median (range)	73.5 (37-81)	62 (32-67)	11 (5-18)	1.5 (0-3)	0 (0-0)	0 (0-0)	0 (0-0)

[1] Single nucleotide variants
[2] Insertions and deletions
[3] Protein-truncating variants. These correspond to variants annotated as nonsense mutations, or frameshift insertions or deletions by GATK4 *Funcotator*.
[4] *ClinVar* annotation of pathogenicity within GATK4 *Funcotator* variant annotation.

Supplementary Table S7. Variants identified through whole-exome sequencing (WES) validation with Sanger sequencing.

Case	Variant Type	Chromosome Number	Position	Reference	Blood	Tissue	R3-Fwd Primer	R3-Rev Primer	Product size (bp)	Sanger Results
2	SNV	12	52949518	C	C	T	GGCCTCTTACCTGGACAGA	TAGGGGTTGGGTGGTTATGG	243	No mutation detected
2	Deletion	3	113294650-113294651	CAA	CAA	deletion	CCCAAGAGGCGTTCAATAAAG	CATCTATTTTGCATCTTCCCACC	373	No mutation detected
3	SNV	12	52949518	C	C	T	GGCCTCTTACCTGGACAGA	TAGGGGTTGGGTGGTTATGG	243	No mutation detected
3	SNV	3	165006783	G	G/A	G/T	GAGCACCTACAATTGGCCAG	ACACGTTTCATTGTAGGGCAC	492	No mutation detected
4	SNV	12	52949518	C	C	T	GGCCTCTTACCTGGACAGA	TAGGGGTTGGGTGGTTATGG	243	No mutation detected
4	Insertion	3	57860849-57860850	TC	TC	TAC	ACAGGGCCAGAATCATTTATGT	AGCGTAACTGTTCCTTCCCA	420	Mutation found in both T & B
4	SNV	2	159386356	G	G	G/T	AATCACCTTGGTCTTTGCCT	TGGTGGTGCAGTTCTTACGA	437	No mutation detected
4	SNV	15	76175545	T	T	T/C	TGGCCCTCTGTACTTTCTGG	CTACCAGTGTCCCCAGCAAG	341	No mutation detected
5	SNV	12	52949518	C	C	T	GGCCTCTTACCTGGACAGA	TAGGGGTTGGGTGGTTATGG	243	No mutation detected
5	Deletion	7	77639409-77639418	GCTGGGACCA	GCTGGGACCA	deletion	TGGACATGATTCAGGGAGCT	ACCTACTTGCCCTGTACATATAC	361	No mutation detected

5	SNV	7	122360949	G	G	G/T	GGAACATCAACATATTTTGCAGC	CCTCAGCTGTCAGTTCATTCA	352	No mutation detected
5	SNV	11	66232302	G	G	G/A	TGTTCCTGCAGATTCCAAGAA	CGTATGAGGAAGCTGAGGTAGA	350	No mutation detected
6	SNV	12	52949518	C	C	T	GGCCTCTTACCTGGACAGA	TAGGGGTTGGGTGGTTATGG	243	No mutation detected
7	SNV	12	52949518	C	C	T	GGCCTCTTACCTGGACAGA	TAGGGGTTGGGTGGTTATGG	243	No mutation detected
